# Identification of bile acid-CoA:amino acid N-acyltransferase as the hepatic N-acyl taurine synthase for polyunsaturated fatty acids

**DOI:** 10.1016/j.jlr.2023.100361

**Published:** 2023-03-22

**Authors:** Samuel A.J. Trammell, Luke F. Gamon, Kamil Gotfryd, Katja Thorøe Michler, Bandar D. Alrehaili, Iben Rix, Filip K. Knop, Pontus Gourdon, Yoon-Kwang Lee, Michael J. Davies, Matthew P. Gillum, Trisha J. Grevengoed

**Affiliations:** 1 Department of Biomedical Sciences, University of Copenhagen, Copenhagen, Denmark; 2 Department of Integrative Medical Sciences, Northeast Ohio Medical University, Rootstown, OH, USA; 3 Department of Pharmacology and Toxicology, Pharmacy College, Taibah University, Medina, Saudi Arabia; 4 Center for Clinical Metabolic Research, Gentofte Hospital, University of Copenhagen, Hellerup, Denmark; 5 Department of Clinical Medicine, Faculty of Health and Medical Sciences, University of Copenhagen, Copenhagen, Denmark; 6 Steno Diabetes Center Copenhagen, Herlev, Denmark; 7 Novo Nordisk Foundation Center for Basic Metabolic Research, University of Copenhagen, Copenhagen, Denmark; 8 Department of Experimental Medical Science, Lund University, Lund, Sweden; 9 Global Obesity and Liver Disease Research, Novo Nordisk A/S, Måløv, Denmark

**Keywords:** bile acids and salts/biosynthesis, bile acids and salts/metabolism, liver, omega-3 fatty acids, peroxisomes

## Abstract

N-acyl taurines (NATs) are bioactive lipids with emerging roles in glucose homeostasis and lipid metabolism. The acyl chains of hepatic and biliary NATs are enriched in polyunsaturated fatty acids (PUFAs). Dietary supplementation with a class of PUFAs, the omega-3 fatty acids, increases their cognate NATs in mice and humans. However, the synthesis pathway of the PUFA-containing NATs remains undiscovered. Here, we report that human livers synthesize NATs and that the acyl-chain preference is similar in murine liver homogenates. In the mouse, we found that hepatic NAT synthase activity localizes to the peroxisome and depends upon an active-site cysteine. Using unbiased metabolomics and proteomics, we identified bile acid-CoA:amino acid N-acyltransferase (BAAT) as the likely hepatic NAT synthase in vitro. Subsequently, we confirmed that BAAT knockout livers lack up to 90% of NAT synthase activity and that biliary PUFA-containing NATs are significantly reduced compared with wildtype. In conclusion, we identified the in vivo PUFA-NAT synthase in the murine liver and expanded the known substrates of the bile acid-conjugating enzyme, BAAT, beyond classic bile acids to the synthesis of a novel class of bioactive lipids.

Omega-3 fatty acids, such as DHA and EPA, are essential fatty acids that can lower cardiometabolic disease risk (1) through regulating fatty acid synthesis and oxidation and resolving inflammation (2). Mammals lack the desaturases necessary to synthesize omega-3 fatty acids and rely upon dietary sources, such as fatty fish and certain plant oils, for these critical lipids. Because of the potential health benefits of omega-3 fatty acids, much interest has been generated in understanding the mechanisms behind their action, including how metabolites of these fatty acids exert their effects.

N-acyl taurines (NATs) are endogenous bioactive acyl-amino acids with the acyl chain conjugated to the beta-amino acid, taurine. Endogenous NATs are found with a variety of acyl chains, and this variation helps determine their biological activity. Oleoyl taurine (C18:1 NAT) is the most abundant species in human plasma and can stimulate glucagon-like peptide 1 secretion to improve insulin sensitivity in mice (3). The DHA-containing NAT (C22:6 NAT) increases in both human and mouse plasma after dietary supplementation of the free fatty acid DHA. In DHA-supplemented wild-type mice, biliary C22:6 NAT concentration increases 26-fold to concentrations that limit lipid absorption and diminish high fat diet-induced fatty liver (4). These functional studies relied upon NAT elevation, but the necessity of NATs in normal physiology or disease remains unknown with the in vivo synthesis enzyme unidentified.

Two candidate enzymes have been identified as having NAT synthase activity in vitro: acyl-coenzyme A amino acid N-acyltransferase 1 (ACNAT1) and 2 (ACNAT2) (5). When purified, these enzymes display NAT synthase activity with saturated acyl-CoAs in vitro. However, ACNAT1 is unable to conjugate arachidonoyl-CoA with taurine (5) and it was only speculated that ACNAT2 could produce PUFA NATs (6). Also, despite the presence of NATs in humans (3, 4), there is no known human homologue of ACNAT1 and ACNAT2, suggesting the existence of other NAT-synthesizing enzymes.

NATs are hydrolyzed by fatty acid amide hydrolase (FAAH) (7), which hydrolyzes several categories of acyl amides, including N-acyl ethanolamides and N-acyl glycines. When FAAH is inhibited, NATs derived from PUFAs accumulate in the liver to a larger extent than less saturated NATs, with arachidonoyl taurine (C20:4 NAT) increasing 166-fold in 3 h (8). This high turnover rate of PUFA-containing NATs suggests biological importance, and their increase after PUFA supplementation led us to the search for the in vivo enzyme responsible for synthesizing PUFA-containing NATs.

Here, we show that human livers synthesize NATs and identify the peroxisome as the site of NAT synthesis in the murine liver. Using metabolomics and proteomics, we identify bile acid-CoA:amino acid N-acyltransferase (BAAT) as the hepatic NAT synthase in vitro and in vivo and demonstrate its preference for synthesis of PUFA-containing NATs. The identification of the enzyme responsible for PUFA NAT synthesis will aid in uncovering the biological function of this novel class of acyl-amino acids.

## Materials and methods

### Mouse care and approval

Wild-type mice protocols were approved by the Danish Animal Experiments Inspectorate and performed according to Animal Research: Reporting of In Vivo Experiments (ARRIVE) standards. Mice were housed in a specific pathogen-free environment on a 12-h light/12-h dark cycle and were maintained on regular chow with free access to food and water unless otherwise specified.

BAAT KO mice and controls were housed in the accredited pathogen-free facility at Northeast Ohio Medical University on a 12 h light/dark cycle. All animal care and use protocols were approved by the Institutional Animal Care and Use Committee of Northeast Ohio Medical University. Mice were fed a high-fat, high-sucrose Western diet supplemented with 0.2% taurocholic acid (Envigo TD.21068) for 6 weeks starting at 10 weeks of age.

### NAT synthase activity assays

Human liver biopsies were obtained from nondiseased liver from five subjects, four female and one male, with mean age 71 years as part of a separate study. The study was approved by the Scientific Ethical Committee of the Capital Region of Denmark (H-18010755) and abides by the Declaration of Helsinki principles. The subjects gave informed consent prior to any trial activity. Mouse and human liver samples were homogenized and diluted in 20 mM Hepes, pH 7.0 with 50 mM NaCl. Protein concentration was determined by BCA assay, and NAT synthase activity was detected using 1–5 μg protein for human liver and 25–100 μg protein for mouse tissues. Linearity of activity was assessed across multiple protein amounts before calculation of rate of activity. Final taurine concentration was 10 mM, and the reaction was initiated by adding 100 μM acyl-CoA, either a single species or equimolar mixture (1 μl in DMSO). The reaction was allowed to proceed for 5 min at room temperature and was stopped by adding four volumes of ice-cold methanol. For N-ethyl maleimide (NEM) treatment, samples were preincubated with 200 μM NEM for 10 min at room temperature before quenching with 200 μM DTT prior to addition of acyl-CoA.

### NAT quantitation

Unless otherwise stated, all solvents used for NAT quantitation were of liquid chromatography-mass spectrometry (LC/MS) grade quality. C15:0 NAT, C18:1 NAT and C22:6 NAT were synthesized as previously described (3).

### NAT synthase activity analysis

After termination of the reactions, samples were centrifuged at 14,000 *g* for 10 min. The supernatants were transferred and processed for either qualitative or quantitative analysis. For qualitative analysis, reaction aliquots were directly analyzed as described below. Quantitation was performed by drying down either 40, 100, or 250 μl of reaction mixture, as indicated in figure legends, in the presence of 5 pmol of C15:0 NAT via speed vacuum with heating at 40°C. The dried reactions were resuspended in 50 μl of milliQ water and centrifuged at 14,000 *g* for 10 min. The supernatants were transferred to Phenomenex Verex autosampler vials (catalog number: AR0-3920-12) and capped with Phenomenex Verex caps (catalog number: AR0-8952-12-M). Standards were composed as previously described (4) for the quantification of NATs in murine plasma. All samples and standards were placed in a Waters ACQUITY FTN AutoSampler maintained at 10°C.

**Fig. 1:**
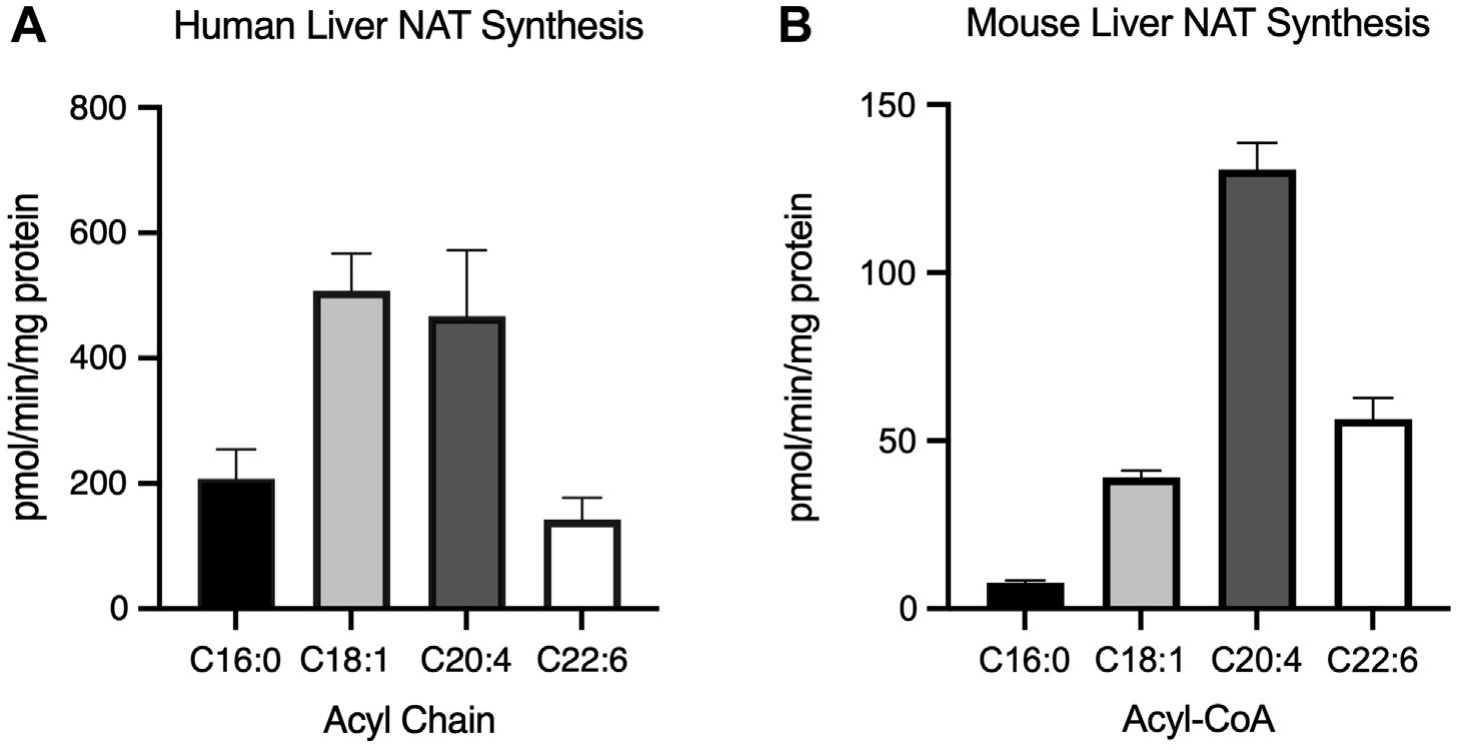
Human liver displays NAT synthase activity similar to mouse liver, despite lack of ACNATs. A: NAT synthase activity in human liver biopsies using an equimolar mixture of acyl-CoAs and 1, 2.5, and 5 μg protein and measured in 250 μl reaction mixture (n = 5). B: Total NAT synthase activity in mouse liver as in A with 50 μg protein and measured in 40 μl reaction mixture (n = 3).

**Fig. 2:**
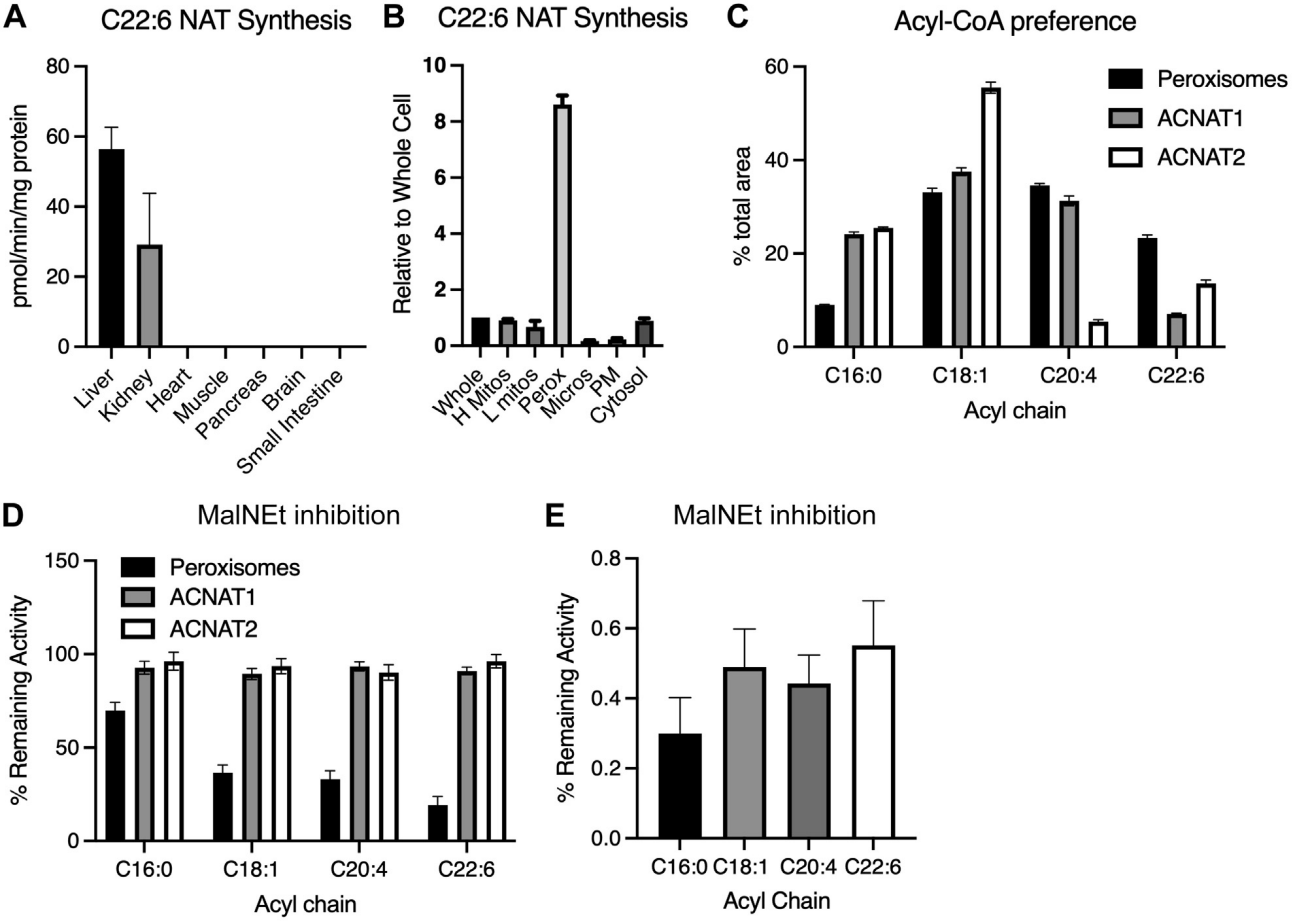
NAT synthase activity is found primarily in hepatic peroxisomes but does not correspond to ACNATs. A: C22:6 NAT synthase activity in mouse tissues (50 μg protein, measured in 40 μl reaction mixture) (n = 3). B: C22:6 NAT synthase activity in hepatic whole cell homogenate and organelle-enriched fractions (n = 9), including heavy mitochondria, light mitochondria, peroxisomes, microsomes, plasma membrane, and cytosol (10, 25, and 50 μg protein). C: Relative acyl-CoA preference in hepatic peroxisomes and lysates of Huh7 overexpressing ACNAT1 or ACNAT2 (10–20 μg protein) (n = 3 independent experiments). D: Relative proportion of activity remaining after NEM inhibition as in C (n = 3 independent experiments). E: Relative proportion of activity remaining after NEM inhibition in human liver, as in Fig. 1A (n = 5).

**Fig. 3:**
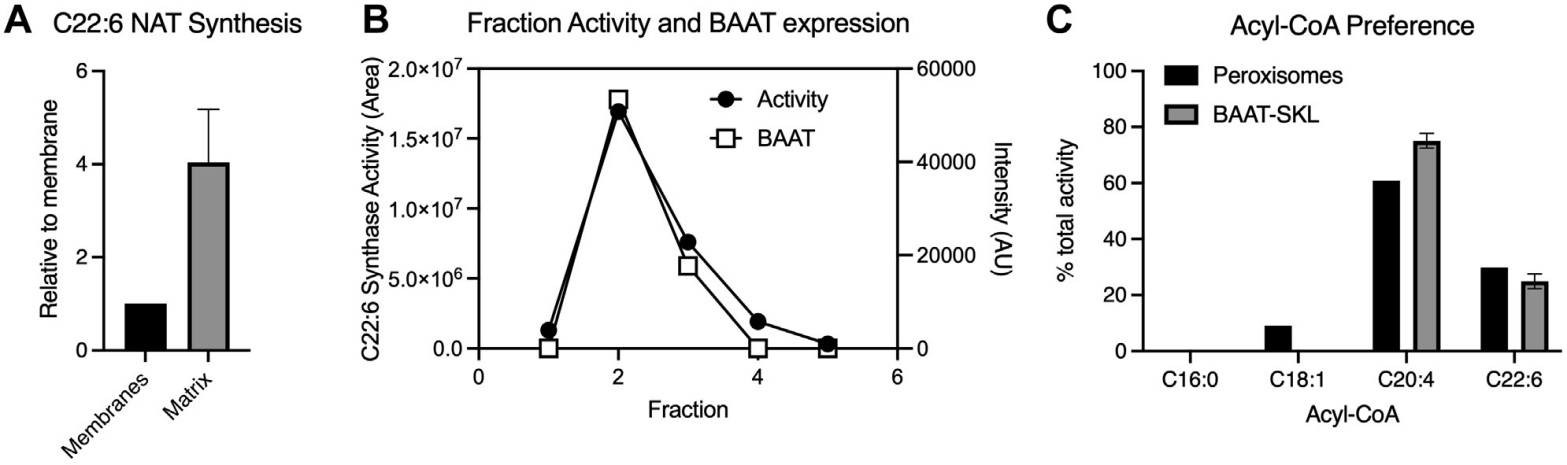
Proteomic identification of BAAT as the likely PUFA NAT synthase. A: C22:6 NAT synthase activity in membranes and soluble fraction from peroxisomes lysed by freezing (20 and 40 μl supernatant or resuspended pellet) (n = 4). B: C22:6 NAT synthase activity (left axis) and BAAT abundance (right axis) in fractions from soluble peroxisomal proteins separated by ion exchange chromatography. Other identified proteins are listed in the Supplementary data (supplemental Fig. S1). Protein intensities are expressed in arbitrary units (AU). C: NAT synthase activity in hepatic peroxisomes and HEK293 overexpressing BAAT-SKL, a peroxisomal-targeted BAAT (9). Cells transfected with an empty vector had no detectable activity, and both peroxisomes and BAAT-SKL had no detectable activity when incubated with NEM (10, 20, and 30 μg protein) (n = 3 independent experiments).

**Fig. 4:**
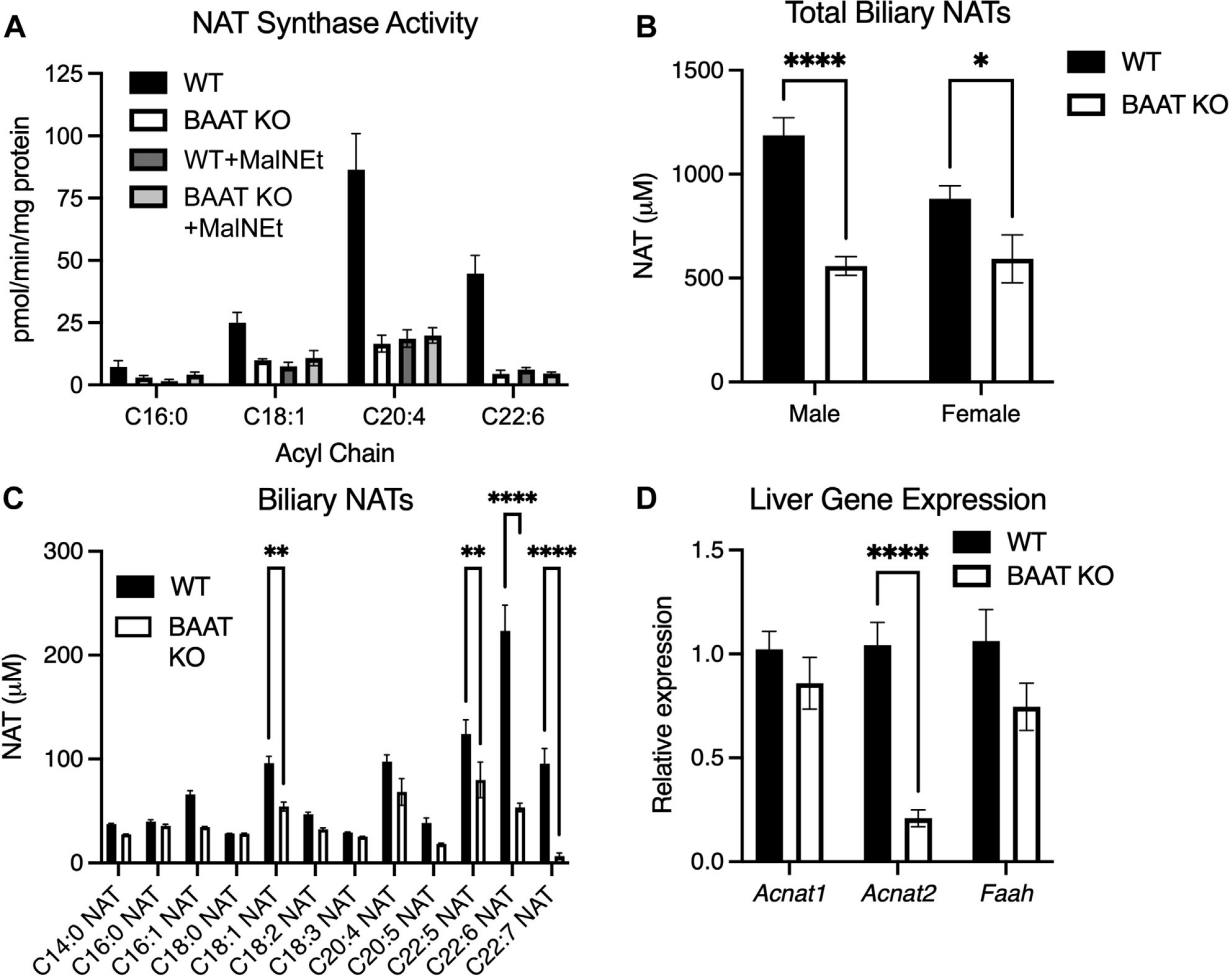
Mice lacking BAAT have low hepatic NAT synthase activity and diminished biliary PUFA NATs. *Baat* KO and littermate controls (WT) were fed a taurocholic acid-supplemented Western diet for 6 weeks. A: Hepatic NAT synthase activity using an equimolar mix of acyl-CoAs, from male mice (25, 50, 100 μg protein, measured in 100 μl reaction mixture) (n = 3). B: Total identified biliary NATs in *Baat* KO mice and WT (n = 5–9). C: Biliary and plasma NAT species from male *Baat* KO and WT (n = 5–9). D: Hepatic gene expression in males (n = 7–9). ∗*P*-value < 0.05; ∗∗*P*-value < 0.01; ∗∗∗∗*P*-value < 0.0001.

NATs (10 μl aliquots) were separated using a Waters Acquity UPLC BEH C18 column (2.1 × 100 mm, 130 Å, 1.7 μm) (catalog number: 186002352) fitted with a guard column (2.1 mm × 5 mm, 130 Å, 1.7 μm) (catalog number: 186003975) with a gradient between 0.01% (v/v) formic acid in water (mobile phase A) and 0.01% (v/v) formic acid in acetonitrile (mobile phase B). The column was equilibrated at 55% B for 20 min before the start of analysis with a flow rate of 0.4 ml min^−1^. The column was heated to 50°C. After injection, the percent mobile phase B was increased to 80% over 6 min. The column was then washed for 3 min at 100% B. Before injection of the next sample, the column was re-equilibrated at 55% B for 2 min. Analytes were detected using a Waters XEVO-TQXS triple quadrupole mass spectrometer operated in negative multiple reaction monitoring mode. The multiple reaction monitoring parameters are described in supplemental Table S1. The following parameters were used on the mass spectrometer: capillary, 3.5 kV; desolvation temperature, 300°C; desolvation gas flow, 800 l h^−1^; cone gas flow, 150 l h^−1^; and nebulizer gas, 7 bar. C18:1-NAT and C22:6-NAT were quantified using bona fide standards normalized to C15:0-NAT signal. Linear curves were fitted using Waters TargetLynx software (version 4.2) with 1/x weighting. C16:0-NAT and C20:4-NAT were quantified using the calibration curves for C18:1-NAT and C22:6-NAT, respectively. The picomolar amount in the sample was calculated by multiplying by the resuspension volume (50 μl) and the dilution factor (5), then dividing this product by the injection volume (10 μl). The picomolar amount was converted to micromolar amounts by dividing by the amount of reaction dried down (40, 100, or 250 μl).

### Murine bile

Murine bile from *Baat* knockouts was prepared, separated, and quantified as described (4). A Bruker Impact II instrument was used for the detection and quantitation of analytes. The instrument was operated using the following conditions: *m/z*, 50–1000; capillary, 4.5 kV; collision energy, 25 eV (optimized for NATs); nebulizer gas flow, 9 l h^−1^; nebulizer gas temperature, 250°C; nebulizer pressure, 1.4 bar; and scan rate, 1 Hz.

### Organelle isolation

Peroxisomes and other organelles were isolated via differential centrifugation. Briefly, mice were fasted overnight and livers were homogenized in homogenization buffer (250 mM sucrose, 5 mM Mops, 1 mM EDTA, 1 mM DTT with protease inhibitors [Sigma]). All subsequent steps were carried out at 4°C. Nuclei and unbroken cells were removed by centrifugation at 600 *g* for 5 min, and heavy mitochondria were isolated from the resulting supernatant by centrifugation at 2,700 *g* for 10 min. The light mitochondria and peroxisomes were spun down at 37,000 *g* for 30 min. The resulting pellet was resuspended in 22.5% Optiprep (STEMCELL Technologies incorporated, catalog number 07820). A gradient was then formed with 27.5% Optiprep, the mitochondria/peroxisome preparation, and 20% Optiprep. The gradient was then centrifuged at 100,000 *g* for 1.5 h, and peroxisomes were collected from the interface of 22.5% and 27.5% Optiprep layers. Microsomes were isolated from the supernatant of the original light mitochondria/peroxisome fraction by centrifuging for 1 h at 100,000 *g*, and the resulting supernatant was considered cytosol. Purity of fractions was confirmed via Western blot. Peroxisomes were lysed via snap freezing in liquid nitrogen, and membranes were then separated from luminal contents via centrifugation at 100,000 *g* for 1 h.

### Protein fractionation

The soluble peroxisomal supernatant was subjected to fractionation using ion exchange (IE) chromatography. Briefly, the sample was diluted 5–10× in the IE buffer (20 mM Hepes pH 7.0) to reduce salt concentration and applied to a 1-mL Resource™ Q anion exchange column (Cytiva, Denmark) equilibrated with the same buffer. Following washes, bound proteins were eluted in 2–60% NaCl gradient applied over the same buffer and fractions of 0.5 ml were collected. Protein-containing fractions were subsequently subjected to SDS-PAGE and enzymatic activity analysis.

### Protein digestion and proteomics

Gel pieces were placed in Eppendorf LoBind protein tubes and washed in 100 μl of NH_4_HCO_3_ (100 mM) for 30 min. The supernatant was removed, and gel pieces were dried by addition of 50 μl acetonitrile. The acetonitrile was removed after ca. 10 min, and the gel pieces were left to air dry for 2–3 min. Proteins were reduced and alkylated by addition of tris(2-carboxyethyl)phosphine (10 mM) and 2-chloroacetamide (10 mM) in 50 μl of NH_4_HCO_3_ (100 mM), followed by incubation at 70°C for 5 min. Gel pieces were washed in 100 μl of 50% acetonitrile:H_2_O, followed by 100 μl of 100% acetonitrile and air dried for 2–3 min. Proteins were digested by addition of 100 μl trypsin (12.5 ng μl^−1^) in NH_4_HCO_3_ (100 mM), followed by incubation on ice for 30 min. Extra trypsin solution was then added, as necessary, to cover the gel piece, which were incubated on ice for a further 90 min, and finally incubated overnight at 37°C.

Samples were subjected to stage-tip solid-phase extraction on C18 discs and analyzed on a Bruker timsTOF Pro mass spectrometer (Bruker Daltonics) in positive ion mode with a CaptiveSpray ion source on-line connected to a Dionex Ultimate 3000RSLC-nano chromatography system (Thermo Fisher Scientific). Peptides were separated on a 25 cm × 75 μm Aurora column (IonOpticks) at 60°C with a solvent gradient over 140 min, using water with 0.1% formic acid (solvent A) and acetonitrile with 0.1% formic acid (solvent B) at a flow rate of 400 nl min^−1^ (0–1 min 2%B; 1–5 min 2–5%B; 5–90 min 5–25%B; 90–100 min 25–35%B, 100–110 min 35–85%B, 110–125 min 85%B, 125–128 min 85–2%B, 128–140 min 2%B). The mass spectrometer was operated in data-dependent acquisition parallel accumulation-serial fragmentation mode (DDA-PASEF) with 1.1 s cycle time and a trapped ion mobility ramp time of 100 ms. MS scan range was set to 100–1700 *m/z*. The fragmentation spectra were searched against the mouse UniProt database (UP000000589, reviewed sequences) plus common contaminants using MSFragger (v3.4) implemented in FragPipe (v17.1). Default settings (peptide length 7–50) were used in the FragPipe data analysis workflow. Proteins were considered candidates if *1*) it is a nonstructural protein, *2*) the protein is detected in the fraction with maximal NAT synthase activity, and *3*) the protein is undetected in fractions without NAT synthase activity.

### Cell culture

Huh7 and Hek293 cells were cultured in DMEM+GlutaMax supplemented with 10% FBS and penicillin/streptomycin. Plasmids containing mouse *Acnat1* or rat *Acnat2* in pCMV6-Entry without a tag (Origene) or empty vector control were expressed in Huh7 cells. Mouse *Baat-SKL* in pRP[Exp]-CMV without a tag (VectorBuilder) or empty vector control was expressed in Hek293 cells. To obtain *mBaat-SKL*, the original gene (NM_007519.3) was altered to change bp1255 from C to A to change the amino acid from Q to K, thus resulting in the C-terminal peroxisomal targeting sequence -SKL. All cells were transfected using Lipofectamine 3000 and collected 48 h later. Cells were scraped into 20 mM Hepes, pH 7.0, 50 mM NaCl with protease inhibitors (Roche).

### Statistics

Data are represented as mean ± SEM. Statistical analysis was performed using GraphPad Prism version 9. Significance was determined by two-way ANOVA with Sidak’s multiple-comparisons test where appropriate. A *P* value ≤0.05 was considered statistically significant.

## Results

### Human liver displays NAT synthase activity similar to mouse, despite lack of ACNATs

We previously established a role of elevated NATs in regulating energy balance and metabolism by inhibiting their degradation and dosing with exogenous NATs (3, 4). In order to establish the necessity of NATs in these processes, it is required to identify their biosynthetic enzymes. ACNATs were previously implicated as NAT synthases (5, 6), but their expression is restricted to rodents with no known human homologue. Because human plasma contains NATs and the levels of omega-3 fatty acid-containing NATs increases with fatty acid supplementation (4), we hypothesized that an additional NAT synthase exists.

NAT synthase activity in human livers was examined using an equimolar mixture of acyl-CoAs (Fig. 1A). Human liver biopsies from individuals without liver disease contained NAT synthase activity with equal substrate preference for oleoyl-CoA (C18:1-CoA) and arachidonoyl-CoA (C20:4-CoA). Whole murine livers were also tested for acyl-CoA preference in NAT synthesis (Fig. 1B). Murine liver NAT synthesis activity was highest for arachidonoyl-CoA followed by docosahexaenoyl-CoA (C22:6-CoA), which was comparable in activity to that of oleoyl-CoA. These data indicate a NAT-synthesizing enzyme(s) is present in both humans and mice.

### NAT synthase activity is found primarily in hepatic peroxisomes but does not correspond to ACNATs

Previous studies indicated that mouse liver has higher C20:4 NAT synthase activity compared with other tissues (10). In order to expand this to C22:6 NAT and additional tissues, we tested NAT synthase activity and acyl-CoA preference in liver, kidney, heart, skeletal muscle (gastrocnemius), pancreas, brain, and small intestine (Fig. 2A). In agreement with previous studies investigating NAT turnover (10), mouse liver and kidney were determined to have the highest NAT synthase activity of the tested tissues. Because the liver has the highest turnover rate of C22:6 NAT and C20:4 NAT (8), we focused on identifying the hepatic NAT synthase for these PUFA-containing species.

Within the liver, enzymatic conjugation of C22:6-CoA to taurine was highly concentrated in the peroxisome-enriched fraction (Fig. 2B), as displayed by peroxisomal NAT synthase activity being 8-fold higher than whole-cell activity. Because the ACNATs are peroxisomally targeted enzymes (5), we compared the substrate preference and sensitivity of the endogenous NAT synthase with overexpressed ACNAT1 and ACNAT2 (Fig. 2C, D). The hepatic peroxisome preparation showed a preference for longer, more unsaturated acyl-CoAs than either ACNAT and was highly sensitive to NEM inhibition, especially for more unsaturated acyl chains. NEM irreversibly alkylates thiol groups, thereby inhibiting enzymes with cysteine residues in their active sites (11). Importantly, ACNAT1 and ACNAT2 were not inhibited by NEM, further suggesting an enzyme other than ACNAT1 and ACNAT2 as the PUFA-NAT synthase in vivo. As ACNATs have an active-site serine, rather than cysteine (5), these enzymes are resistant to NEM treatment. In addition, the NAT synthase activity in human liver biopsies was reduced approximately 50% by NEM (Fig. 2E). These data indicate that the in vivo hepatic synthase for PUFA-containing NATs localizes to peroxisomes and contains an active-site cysteine.

### Proteomic identification of BAAT as the likely PUFA NAT synthase

To identify the in vivo enzyme responsible for NAT synthesis, we turned to an unbiased approach using IE chromatography and bottom-up proteomics. We determined that the NAT synthase is a soluble, non-membrane-bound peroxisomal protein (Fig. 3A). The soluble protein fraction was separated by IE. Fractions containing NAT synthase activity were identified, and proteins in active fractions were separated using SDS-PAGE. Proteins in the bands were examined using bottom-up proteomics based on reverse-phase high-resolution mass spectrometry. A total of 51 proteins were identified with this approach. Structural proteins and proteins absent in fraction 2, which contained most of the C22:6 NAT activity (Fig. 3B), were removed from further consideration. Of the 14 remaining proteins, only three proteins were present in all C22:6 NAT active fractions (Fig. 3B and supplemental Fig. S1). Enolase 1 (ENO1) is a glycolytic enzyme that converts 2-phosphoglycerate to phosphoenolpyruvate (12, 13). Carboxylesterase 1d (CES1D) is part of the serine hydrolase superfamily with broad substrate specificity, including fatty acyl-CoAs (14). However, as with ACNAT1 and ACNAT2, CES1D depends upon an active-site serine, not a cysteine. This left one candidate: BAAT (Fig. 3B). Through a catalytic cysteine (5, 11), BAAT catalyzes the conjugation of CoA-bound bile acids with taurine or glycine and can also conjugate acyl-CoAs with glycine (5, 11, 15). It is located in both peroxisomes and cytosol with most bile acid-conjugating activity in peroxisomes (16). Our data show that BAAT and liver-derived peroxisomes display similar acyl-CoA preference and NEM sensitivity (Fig. 3C), indicating that BAAT is a likely in vivo NAT synthase candidate.

### Mice lacking BAAT have low hepatic NAT synthase activity and diminished biliary PUFA NATs

To test whether BAAT is the in vivo NAT synthase, liver from wild-type (WT) and *Baat* knockout (KO) mice (9, 17) were analyzed for activity (Fig. 4A). NAT synthase capacity decreased by up to 90% in *Baat* KO mice compared with WT, with the greatest losses of activity detected for C22:6 NAT and C20:4 NAT. Importantly, NEM treatment decreased activity in WT livers to that of the *Baat* KO and did not appreciably lower activity within *Baat* KO livers, suggesting BAAT is responsible for nearly all of the measured activity in the tissue. We have previously shown that murine bile is enriched in NATs (4) and this enrichment is likely due to synthesis in the liver. Loss of BAAT lowered total biliary NATs (Fig. 4B), especially polyunsaturated NAT species, such as C22:6 NAT, which was 76% lower in the *Baat* KO bile (Fig. 4C). These data indicate that BAAT is the primary hepatic NAT synthase and is responsible for biliary polyunsaturated NAT production. Expression of hepatic *Acnat1* and *Acnat2* was not elevated in *Baat* KO murine liver (Fig. 4D), indicating no compensation by these enzymes. Despite lower expression of *Acnat2*, NEM-resistant NAT synthase activity is not lower in the *Baat* KO livers. Expression of the NAT degrading enzyme, *Faah*, was likewise unaltered (Fig. 4D). Together, using unbiased techniques and in vivo models, we identified a novel role for BAAT as the hepatic PUFA NAT synthase.

## Discussion

NATs are evolutionarily conserved from marine sponges (18, 19), sea urchins (20), and crayfish (21) to mice and humans (3, 4). The acyl-chain profile varies between species and within murine tissues (8), and PUFA-containing NATs are enriched in the murine liver and bile. This specific acyl-chain profile is likely indicative of enzymatic preference or location-based substrate availability and may have important implications for biological function in each species. Despite the clear conservation of NAT synthesis across the animal kingdom and enrichment of essential fatty acyl chains, little is known about their synthesis and biological importance.

Prior to this work, the NAT degradation pathway through FAAH has been well studied, but synthesis pathways have been largely unexplored. To understand the biological role and necessity of NATs, identifying the NAT synthase enzyme(s) is critical. By using C22:6-CoA as a substrate in our identification of the hepatic NAT synthase, we directed our focus to an enzyme that prefers polyunsaturated acyl-CoAs. ACNAT1 and ACNAT2 were previously implicated as NAT-synthesizing enzymes using saturated acyl-CoAs. Whether these enzymes conjugated unsaturated acyl-CoAs to taurine remained unknown. Given that C22:6 NAT is elevated in response to omega-3 fatty acid supplementation (4) and has a rapid turnover rate in liver (8), it was likely that this NAT species was enzymatically synthesized and preferred by a hepatic protein. By using NEM as a chemical inhibitor, we ruled out the ACNATs as the major NAT synthase, especially for PUFA-containing NATs. Because of our PUFA-centered approach, we identified BAAT to be the enzyme responsible for hepatic synthesis of PUFA-containing NATs, which was confirmed in both overexpression and knockout models. Interestingly, purified rat BAAT has been reported to have no conjugating activity with palmitoyl-CoA and taurine (22), agreeing with our observations that BAAT prefers PUFA acyl chains to saturated acyl chains (Figs. 1, 2C, 3C and 4A). This finding enables future studies to determine the importance of synthesis of these NATs in omega-3 fatty acid supplementation and establish their roles in lipid metabolism.

The newly identified NAT synthase, BAAT, is well known as the only bile acid-conjugating enzyme in the rat (22) and human (23). In these reactions, a steroidal bile acid conjugated to coenzyme A is conjugated to either taurine or glycine, which increases the hydrophilicity of the bile acid and improves its ability to emulsify fats. In the animal kingdom (24), BAAT conjugates bile acid-CoAs composed of 24 or 27 carbons containing various numbers of hydroxyl groups also varying in their position around the steroidal core of the bile acid. The position and number of hydroxyl groups determines the hydrophilicity of the bile acid (25), which affects their amide conjugation with increasing hydrophilicity decreasing conjugation (24, 26). Analogous to bile acids, PUFAs charged with CoA tend to be higher in carbon content (≥18) (27) and increase in hydrophilicity and rigidity, not with hydroxyl groups, but with the number of double bonds (28). Future study is warranted to understand the structure-activity relationship between the physicochemical properties of acyl-CoAs and taurine conjugation catalyzed by BAAT.

Loss of function of BAAT in humans leads to cholestasis in children and poor fat and fat-soluble vitamin absorption (29). Mouse knockouts of BAAT phenocopy the human disease and experience alterations in the gut microbiome (17, 30). To date, the role of NATs in the pathology of BAAT loss of function is unexplored. Interestingly, elevating C22:6 NAT impairs lipid absorption (4), but the effects of low biliary C22:6 NAT are unknown. Omega-3 and omega-6 fatty acids are clearly enriched in biliary NAT acyl chains (4) through an apparent substrate preference of BAAT. It is possible that taurine conjugation to these essential fatty acids regulates their levels via an unknown mechanism. Further studies would appear to be warranted to investigate how impaired hepatic NAT synthesis in the BAAT KO affects PUFA metabolism.

As observed previously (10) and confirmed here (Fig. 2A), other tissues display NAT synthase activity. As BAAT is expressed exclusively in the liver (31), additional synthase enzymes likely exist in other tissues. These enzymes may account for the residual NATs found in the bile of the *Baat* KO animals, especially for shorter or more saturated species. Importantly, PUFA-type NATs were the main species that were lower in the *Baat* KO bile. The peroxisomal location of BAAT likely puts it in contact with higher amounts of unsaturated acyl-CoAs, specifically C22:6- and C20:4-CoAs, as peroxisomes are specialized to metabolize very-long-chain acyl-CoAs and shorten these acyl-CoAs before import into mitochondria for complete oxidation. This difference in substrate availability alone could have driven apparent in vivo synthesis, but when presented with saturated, monounsaturated, and polyunsaturated acyl-CoAs in equimolar concentrations, BAAT shows a distinct preference for polyunsaturated acyl chains. This preference is apparent in both overexpressed peroxisomal BAAT and *Baat* KO livers and WT livers treated with NEM to inhibit BAAT activity.

Overall, this work identifies BAAT as a hepatic NAT synthase with a preference for polyunsaturated acyl-CoAs in both in vitro systems and an in vivo model. This identification opens the door to future studies on the role of NAT synthesis in PUFA metabolism, as well as the roles of NATs in pathologies, such as fatty liver disease and diabetes, thus establishing the biological roles of these conserved lipids.

## Data availability

All data are available upon reasonable request to the corresponding author, Trisha J. Grevengoed at the University of Copenhagen (email: grevengoed@sund.ku.dk).

## Supplemental data

This article contains supplemental data.

## Conflict of interest

The authors declare that they have no conflicts of interest with the contents of this article.
