# Supplementary material for: Identification of bile acid-CoA:amino acid N-acyltransferase as the hepatic N-acyl taurine synthase for polyunsaturated fatty acids
Source: J Lipid Res. 2023 Mar 22;64(9):100361. doi: 10.1016/j.jlr.2023.100361 (PMC10470208; doi:10.1016/j.jlr.2023.100361)
Supplement: Supplemental Figure S1 and Table S1 [file mmc1.pdf]

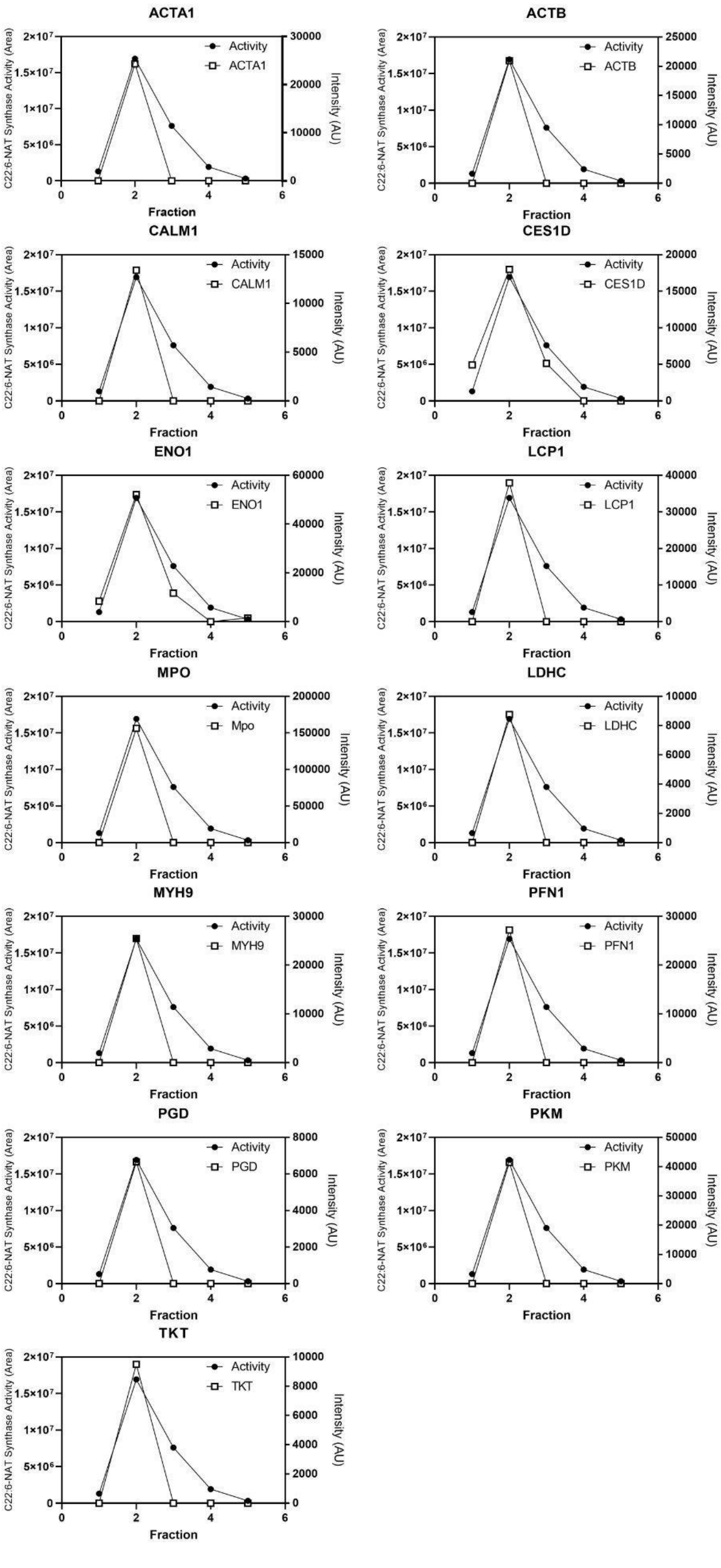

ACTA1
Activity
ACTA1
ACTB
Activity
ACTB
CALM1
Activity
CALM1
CES1D
Activity
CES1D
ENO1
Activity
ENO1
LCP1
Activity
LCP1
MPO
Activity
Mpo
LDHC
Activity
LDHC
MYH9
Activity
MYH9
PFN1
Activity
PFN1
PGD
Activity
PGD
PKM
Activity
PKM
TKT
Activity
TKT
C22:6-NAT Synthase Activity (Area)
Intensity (AU)
Fraction

Supplemental Figure 1. Proteins identified in the fractions containing NAT synthase activity. C22:6 NAT area is plotted on the left y-axis, whereas protein intensity is plotted on the right y-axis. Units are expressed in arbitrary units.

**Acyl-NAT**	**Precursor Ion *m/z*:**	**Product Ion *m/z*:**	**Cone Voltage (V)**	**Collision Energy (eV)**
C14:2 NAT	330.2	80	10	35
C14:0 NAT	334.2	80	10	35
C15:0 NAT	348.2	80	10	40
C16:5 NAT	352.2	80	10	35
C16:1 NAT	360.2	80	10	35
C16:0 NAT	362.2	80	10	40
C16:5-OH NAT	368.2	80	25	50
C18:4 NAT	382.2	80	10	35
C18:3 NAT	384.2	80	10	35
C18:2 NAT	386.2	80	80	40
C18:1 NAT	388.3	80	80	40
C18:0 NAT	390.3	80	80	40
C19:1 NAT	402.3	80	70	40
C19:0 NAT	404.3	80	70	40
C20:6 NAT	406.2	80	70	40
C20:5 NAT	408.2	80	70	40
C20:4 NAT	410.2	80	70	40
C20:4-OH NAT	426.2	80	25	50
C22:7 NAT	432.2	80	70	40
C22:6 NAT	434.2	80	70	40
C22:6-OH NAT	450.2	80	70	40

Supplemental Table 1. Multiple reaction monitoring parameters used for the detection and quantification of NATs in the activity assays.
